# The intertwining of reconciliation and displacement: a lifeworld hermeneutic study of older adults’ perceptions of the finality of life

**DOI:** 10.1080/17482631.2020.1799588

**Published:** 2020-08-07

**Authors:** Lina Palmér, Maria Nyström, Gunilla Carlsson, Catharina Gillsjö, Irene Eriksson, Ann-Charlotte Dalheim-Englund

**Affiliations:** a Faculty of Caring Science, Work Life and Social Welfare, University of Borås, Borås, Sweden; b School of Health and Education, University of Skövde, Skövde, Sweden; c College of Nursing, University of Rhode Island, Kingston, RI, USA

**Keywords:** Existential matters, existential caring science, caring dialogue, lifeworld hermeneutics, finality of life, older adults

## Abstract

**Purpose:** This study aimed to explain and understand the existential meaning of the finality of life from the perspective of healthy older adults.

**Method:** Participants were recruited from a major project on older adults’ life situations. They were interviewed about their thoughts on the end of life, and their responses were interpreted using a lifeworld hermeneutic approach.

**Results:** The findings showed that thinking about the inevitable finality of life involves feelings of liberation, frightening thoughts, a comforting promise of something beyond death, acceptance of the concept of death as a companion in life and a desire to live. Philosopher Simone de Beauvoir’s existential ideas about ageing and death were then used to further explain and understand the meaning of the finality of life and to support a comprehensive understanding. de Beauvoir suggests that when the temporal horizon of existence shrinks, one lives closer to the finality of life. For a comprehensive understanding, attributing meaning to the finality of life required the intertwining of reconciliation and displacement. The interpretations were further discussed using ideas from the fields of existential philosophy and caring science in order to develop a basis for caring practice.

**Conclusions:** The conclusions suggested that professional health care for older adults would benefit from a lifeworld-led caring science approach that includes readiness for a caring dialogue that focuses on existential issues.

## Background

The ageing process varies in terms of health and well-being, but most older adults in the Western world can look forward to increased life expectancy. Research on later life covers two main areas. One of these concerns biological ageing with an increased risk of ill health and impaired functions (Kontos, 1999; Lennartsson et al., 2014), while the other relates to social and existential matters. The latter involves investigations of life without a professional identity (Dalheim-Englund et al., 2018; Gynnerstedt, 2011; Nordenfeldt, 2003) and the final part of life when the need for support and help is evident (see for example Mitzner et al., 2009). Although it seems reasonable to believe that life for individuals during old age is just as diverse as for those of younger age, the finality of life may be neglected earlier in life. Generally, one acknowledges the finality of life by being part of a generation that successively disappears (se for example Dalheim-Englund et al., 2018; Palmér et al., 2018).

Humans are aware of their own mortality, and the importance of this existential issue means that research on people’s thoughts and feelings about death will never become irrelevant. More than 40 years ago, Becker (1973) suggested that the awareness of our mortality is often repressed to allow us to function effectively in daily life and as a defence against the anxiety caused by the awareness that death is inevitable.

Researchers have explored possible differences across age groups in relation to concerns about death. Kalish and Reynolds (1977) found that although older adults think more about their own death than younger individuals, they seem to be less afraid of death than younger people. Russac et al. (2007) confirm this age-related decline in death-related anxiety, which they suggest is relatively high in young adulthood. According to Maxfield et al. (2007), older adults respond differently to questions about death than younger adults. In a later study, Maxfield et al. (2014) found that older adults often respond to reminders of their mortality by becoming more prosocial. Chopic (2017) suggests that lower levels of death anxiety are related to social relationships, which can function as emotional regulators for individuals as they age.

However, the picture is not entirely clear. Scott and Weems (2013) emphasize that death is an individual concern that signals the end of one’s existence, which can cause considerable anxiety, even in old age. Twenty years ago, Fortner and Neimeyer (1999) summarized the literature on death anxiety in older adults and concluded that weak integrity, as well as physical and psychological problems, were predictive of higher levels of death anxiety. Depaola et al. (2003) explored the relationship between death anxiety and personal anxiety about ageing in a group of 197 older men and women. They found correlations between negative attitudes towards older adults and personal anxieties about ageing, death and, in particular, fear of the unknown. Older women scored higher on a “fear of death” scale than older men, and older Caucasians scored higher than older African Americans.

Thinking about the finality of life can also arouse feelings of not wanting to live anymore. Conwell et al. (2011) argue that the increase in suicide rates among older adults requires the implementation of urgent suicide prevention measures tailored to this age group. Rossom et al. (2018) find suicidal ideation in older adults to be strongly associated with severe depression and recommend that all older adults with suicidal ideation be screened for depression. For the same reason, Cahoon (2012) developed a guide in the form of a checklist that health care professionals can use to recognize depression in older adults. Mitty and Flores (2008) discuss the risk factors that should be considered in geriatric care, including stressors such as loss, recent terminal diagnosis, deepening disability, bereavement and relationship disruption.

The older we become, the closer death is, but, according to Van Orden et al. (2014), there is no reason to believe that older adults generally think about death and dying. Palmér et al. (2018) found that older adults who reflect on the last part of life during in-depth interviews talk freely about how to reach a worthy end of life and, in particular, how to avoid dying with extreme suffering. Such thinking about a worthy end of life entails acknowledging death. It is also worth noting that end-of-life care not only includes the alleviation of pain and suffering but can also include treatments related to cognition, mobility and other functions (Koller & Rockwood, 2013).

The current study is part of a Swedish project based on a lifeworld approach to healthy older adults’ life situations. Previous research has explored life after retirement (Dalheim-Englund et al., 2018) and the meaning of growing old (Palmér et al., 2018). Dalheim-Englund et al. (2018) found that freedom after retirement is highly valued but also threatened by an increased risk of ill health. Palmér et al. (2018) found that the fact of being close to the end of life raises questions about a meaningful final stage of life.

A patient who wants to talk about the finality of life needs a caregiver willing to participate in a dialogue about existential issues. A caring dialogue with such a theme requires both courage and knowledge of what the final stage of life can be like. The current study therefore aims to explain and understand the existential meaning of the finality of life from the perspective of healthy older adults who are not yet there but know that the final stage of life is approaching.

## Approach and method

This study uses a lifeworld hermeneutic approach to fulfil the aim of studying existential issues empirically (Dahlberg, Dahlberg, et al., 2008; Nyström, 2017). In this study, the phenomenon of the “existential meaning of the finality of life” is in focus. A lifeworld hermeneutical approach is closely connected to the development of modern hermeneutics introduced by Gadamer (1996), who was, in turn, inspired by Husserl’s theory of the lifeworld (Husserl, 1970/1900). In accordance with Gadamer’s hermeneutic philosophy, all efforts are made to conduct the analysis with optimal openness by adopting a critical attitude to one’s own pre-understanding. The open attitude was supplemented with Ricoeur’s (1976) more method-oriented ideas on hermeneutics in order to tentatively explain latent meanings in the data, and to use theoretical support in the comprehensive understanding.

## Participants and data collection

Participants were chosen via a questionnaire on older adults’ lifestyles for another study in the above-mentioned project on older adults’ life situations, which has not yet been published. That study included questions on demographic data, medical history, and family relationships and used the following validated questionnaires: the sense of coherence scale (SOC-13), sense of coherence scale emotional (SOC-E) and sources of meaning profile (SOMP) (Antonovsky, 1993; Flensborg-Madsen et al., 2006; Langius & Björvell, 1993; Reker, 1996). After completing the questionnaire, the participants were asked if they wished to participate in the current part of the project. Those who agreed supplied their telephone number. Of these, participants were chosen who seemed to be able to contribute to a varied and rich dataset, not only according to variations in age, residence and gender, but also according to variations in SOC scores. The selection took place in two medium-sized Swedish cities surrounded by smaller towns and rural areas. Twenty-four seniors were telephoned to confirm whether they still agreed to be interviewed and if they still experienced their health as good. Those who said yes were chosen for the study. Eighteen persons, comprising seven men and 11 women aged between 72 and 91 (M = 78), took part in the study. Data were collected through individual lifeworld interviews (Dahlberg et al., 2008), during which the following questions were asked:

- What does it mean for you to be older?
- What is most important in your everyday life?
- What does it mean to be healthy in old age?
- What do you think about the end of life?

A lifeworld interview is oriented towards a particular phenomenon and focuses on the meanings attributed to lived experience. The participants were encouraged to reflect on and describe their personal thoughts and feelings about each question. Flexible, probing questions were asked to direct the interviewees to reflect upon matters not immediately described and to deepen their descriptions. The interviews, which lasted about an hour, were audio-taped and transcribed verbatim. The analysis focuses mainly on responses to the last question about the end of life. Responses to the other questions are analysed in other publications (Dalheim-Englund et al., 2019; Palmér et al., 2019).

## Data analysis

The analysis is characterized by a movement back and forth between a first whole (becoming familiar with the whole set of data), parts (suggesting how to explain and understand different aspects of data) and a new whole (a main interpretation that covers all aspects of the new understanding). Initially, all interviews were read several times to become familiar with all data relevant to the aim of the study. As with the entire research process, this reading required the adoption of an open, flexible and reflective attitude, with the least possible influence from pre-understanding and without any predefined theoretical inspiration. The concrete interpretative analysis began when the first whole (all the interviews considered together) felt familiar. Units of meaning, which are parts of the text that carry meaning related to the phenomenon of the finality of life, were identified and marked. When all the meaning units and meanings had been identified and structured, they were compared to identify patterns of meaning about the finality of life that belonged together. Similarities in meanings were considered together to interpret themes of meaning. A theme was interpreted when a general or common underlying meaning appeared to exist. Five interpreted themes of meanings were developed, which should be considered as suggestions for underlying meanings in different aspects of the research phenomenon “finality of life”.

Finally, a further comparative analysis gave rise to a comprehensive understanding, i.e., a main interpretation, that linked all previous interpretations together. To further explain and understand the existential meaning related to the research phenomenon, the open approach was replaced for the benefit of philosopher Simone de Beauvoir’s existential ideas about ageing and death (De Beauvoir, 1996). Her philosophy was used to support and develop the comprehensive understanding. de Beauvoir’s ideas are briefly described in the Findings section.

To strengthen validity, the themes that emerged and the subsequent interpretations were compared with the interview data to ensure that they reflected the research phenomenon rather than any of the researchers’ pre-understandings of it. Any contradictions between the interview data and the interpretations were explored, and the researchers ensured that there was a strong connection between the data and interpretations. Where discrepancies were found, an interpretation was reconsidered, re-worded or omitted (Dahlberg, Dahlberg, et al., 2008; Nyström, 2017).

However, it is worth noting that each interpretation represents one of several ways of understanding a meaning that may be partially or completely hidden in statements from the participants. To enable a critical reading that invites readers to develop their own understanding, we have strived for transparency. The excerpts from data shown are representative of an aspect of the entire data set.

## Ethical considerations

The ethical considerations for the study were identified according to the principles set out in the Declaration of Helsinki (World Medical Association, 2013). Brief written information about the study was provided when the participants were first asked whether they were willing to be interviewed after completing the initial questionnaire mentioned above. When those selected for participation were contacted by telephone, they were informed in more detail about the aim of the study and assured that participation was voluntary and it was possible to withdraw at any time. All who were chosen for the study were assured of confidentiality.

## Findings

The first part of the findings consists of the five interpreted themes, each of which corresponds to an aspect of the research phenomenon. The interpreted themes suggest that the finality of life can be understood as a liberation, a frightening thought, a comforting promise of something beyond death, acceptance of the concept of death as a companion in life and a desire to live. These interpretations are presented below with extracts from the data. As part of the transcription from spoken language, the extracts have been edited for clarity, but their meaning has been retained. Following this, a comprehensive understanding of all themes at a deeper, abstract level is presented.

## A liberation

Ageing entails awareness of the fact that the finality of life approaches, even if a person is still in good health. This insight appears to generate a wish that life will end before the body’s functions are so impaired that the person becomes successively more dependent on other people’s care and support:

I hope my life will not be too long. I am happy with my life now, so I think it would be nice if it stopped now before it comes to ailments and dementia. It would be nice for my children if I don’t get that old.

If I am worried about anything, it is that I should be so bad that I cannot cope with my life myself. Then it is restful to know that it will end sometime. I’m not afraid of it.

Being in the final part of life means that death can occur at any time. One aspect of this insight appears to present a kind of security, because death can be considered a form of protection against unbearable suffering. Life is meaningful only when it is lived as wished. When this is not possible, life may be considered meaningless and not worth living. Death also liberates family members from shouldering the burden of care if an older parent becomes ill.

Hence, the knowledge that life will end seems to generate a sense of freedom, in the sense that death can come as a liberator when it is the only thing that can put an end to illness, suffering and dependence on others. However, the idea of death as a liberator implies a desire to live life to the fullest before suffering limits all possibilities to live the life that is desired. Thus, liberation means the possibility of avoiding a life that a person no longer wants to live.

## A frightening thought

The absoluteness of death can also be frightening in the sense that the temporality of life is uncontrollable. Oneself or a beloved person can at any time be affected by a serious illness that can only end in death:

I try not to think about it. The thoughts come when one hears that someone familiar has become ill. I feel that now it is a matter of living out life when you can; this [a disease] is dangerous.

Frightening thoughts of death can become more tangible after an illness that reminds one of death. To continue to live, thoughts of death must be postponed and repressed:

I live out life as much as I can until something happens to me (a disease or illness), which it certainly will in the future; it will happen to everyone. I have a little inherent fear of this old age.

One can understand that life is finite, but I postpone death.

Thus, the anticipation of the finality of life and death can have an impact that indeed is frightening. When different and anxious feelings become dominant, the only way to handle this might be to displace thoughts of death. Making sense of everyday life, then, includes a strategy to think about something else. Yet this does not always help, when ill health becomes a fact for oneself or a closely related person. Thinking about the possibility of loneliness makes it ever so difficult to maintain an evasive and distant attitude towards death, making the finality of life frightening in a double sense. It entails both the risk of dying oneself and of losing a beloved person.

## A comforting promise of something beyond death

Belief in something beyond death implies that there is a life after death, and that physical death is not the end of an individual’s experience. It can be comforting in moments of despair and a way of avoiding death anxiety:

I don’t think everything is over with death. Just like you can have contact with those who are dead today, they [the dead] are somewhere and follow us. That certainty makes me feel safer and not afraid.

The belief that life is predetermined by a greater authority can be calming and grants hope. Trust in something beyond death can be linked to a religious belief that can provide a sense of security. In God, one can see support and power, which can make life easier and create a sense of belonging:

Obviously, there is a higher power of some kind.

I have my faith in God. Without that, I wouldn’t be able to reconcile my life. I have had a sick husband all my life who died four years ago. And then, probably the most important thing is that I can trust my faith.

Awareness of the finiteness of human existence can thus involve a comforting promise about something beyond death, indicating that humanity is bigger than one’s own life. Such a promise brings the security that life continues in another form when life on earth is over. This view of life can also entail a conviction that it is possible to have contact with dead people in a spiritual or transcendent sense. It can also include a certainty that the finiteness of human existence does not lie in human hands but in those of a greater authority beyond human control. Such an authority can be understood in spiritual terms, which can be connected to religion or other forms of spirituality.

## A desire to live

Approaching the finality of life means that life cannot be taken for granted anymore. Death interrupts life, and it can happen at any time. To recognize that this interruption means acknowledging that everything that is experienced as meaningful will be lost, which in turn can make life even more valuable:

I am aware that something can come, both for my husband and for me; nothing is given anymore.

When thoughts about the finiteness of life are present, a desire emerges to manage the present day in such a way that one will feel satisfied with what has been accomplished at the end of the day. Life is considered a valuable experience be managed in a proactive manner for as long as possible. Such thoughts also generate a desire to plan for the future and not to relinquish the idea that life proceeds:

I try to do something positive every day because I am aware that my time is limited. I want to be able to lay down at night and say this was, after all, a good day.

I do not postpone anything. I do everything I want to do immediately, and I always plan ahead. But I know deep inside that it may not go on forever.

It is not necessarily the fact of dying or of being dead that is frightening, but rather the fact that one will not be allowed to live anymore. Confrontation with one’s mortality thus seems to increase the desire to live. The fear and sorrow of no longer being able to take part in the living world create a willingness to live in the present for as long as possible:

I don’t feel afraid to die, but I’m afraid I won’t be able to do and cope with everything I have left to do.

The desire to live also includes a wish to live on in the memories of those who survive after one’s own death. This desire motivates people to leave behind permanent memories in the form of photographs, maps and books that will remind others about their existence:

They [the children] will never understand. They will never remember me. If I am dead, then these memories are gone. I will fix all the pictures, all the cards we have of our family.

Awareness of the finiteness of human existence can thus generate a wish to live out the life that remains as much as possible. To make sense of the final stage of life includes finding a way to process thoughts of death and attributing meaning to both life and death. This process enables a person to make sense of one’s life and its finality. Death is made meaningful by ensuring that one will exist in the memories of others.

## Acceptance of the concept of death as a companion in life

Consciously thinking about the finality of life means not pushing away thoughts on death. Daring to talk about it, and acknowledging the finality of life, can be helpful in accepting the fact that death is inevitable:

Many say they do not think of death. But I think it is necessary to do so. You learn to accept it.

The experience of losing a loved one deepens a person’s insight into the finiteness of human existence. This experience can produce the terrible insight that another loved one could be lost as well. Yet thinking about the death of a beloved person can also become an experience that enriches one’s own life over time, and this can be expressed as follows:

I have seen so many who have passed away. When my mother died, it was completely terrible at first. But then I understood that it was actually not my death, and that was quite a comforting thought. One can enjoy one’s life! I actually have the responsibility to manage my own life.

The finiteness of human existence may also have been apparent in one’s previous profession during the period in which they worked. Through this, an awareness of the existential dimensions of life may have been processed in everyday life. To rest in the factuality of death and to regard one’s own finiteness as a mystery can be a soothing way of managing the finality of life:

In my previous profession, I thought a lot about death. Existential issues can never conform to rules. Every person is unique. The meaning of life is the greatest question of existence.

Still, since I have had so much to do with death in my job, death may not be as scary, just another side of being human.

Hence, thoughts of death can represent a companion in one’s own life. Such an attitude helps to keep the finality of life visible as an existential fact. Understanding the reality of one’s own death may also increase when one is close to a person who is dying. The death of another person then seems to attribute meaning to death. For the finality of life to be a companion in life, the courage to remain and be present in such a situation appears to be necessary. Being present at the death of another person enables one to have existential thoughts about one’s own life and death, as well as the life and death of those to whom one is closely related. To attend to the death of others make one feel the presence of death, which can be calming, as it enables one to begin to work through thoughts of one’s own death.

### Comprehensive understanding

Temporality is the common denominator in the interpreted themes regarding awareness of the finality of life for older adults experiencing themselves as healthy and feeling well. In order to feel health and well-being in old age, it is particularly important to come to terms with the past, take advantage of the present and the time that remains and give this time a meaning.

Simone de Beauvoir’s existential philosophy inspired the interpretative work of explaining and understanding the phenomenon of “existential meaning of the finality of life”, which in this study is interpreted as an intertwining of displacement and reconciliation of old age and death. In *The Coming of Age*, De beauvoir (1996) deals with the existential dimensions of old age and death, being-in-the-world and the lived experiences being old. de Beauvoir means that old age involves temporality, or more precisely, a changed relationship with time, with the world and with one’s own history. An older adult’s relationship with the past, experience of the present and approach to the future are different from those of younger persons. An older adult undergoes an alteration in their approach to the world in which there is a risk to get stuck in memories from the past. If memories takes over life, the older adult have difficulties living in the present and cannot contemplate death. de Beauvoir means the present and the past are intertwined and influence the choices and experiences regarding old age and death. This is congruent with the interpretations in this study, wherein older adults experiencing themselves as healthy and living a life of well-being can look back on a life with which they are satisfied and/or can reconcile themselves. This makes it possible to also approach life as infinite and give this infiniteness a meaning. Furthermore, de Beauvoir means that older adults themselves have the freedom to overcome the challenges of being old and continue to pursue goals and projects that give existence meaning

In congruence with de Beauvoir’s ideas about temporality, a more intrusive awareness of the finality of life in old age emerges in the findings of this study. Despite the presence of frightening thoughts, death is interpreted as a liberation and a companion, which appears to strengthen the desire to live the life that remains to the fullest. Although de Beauvoir’s philosophy does not relate to any religious belief, the interpretation of the finality of life as a comforting promise of something beyond death also links to the idea of making the final part of life and even the eventuality of death meaningful.

The increasing awareness of death can, according to de Beauvoir, also be understood as the possibility of authentic human existence, thus living a more authentic life in old age than earlier in life. This can be seen even in the empirical findings of the present study. In de Beauvoir’s philosophy, the biological fact of death has neither a determined nor a universal meaning in itself. Rather, it is the older adult’s thoughts and feelings about death while they are still alive and in relation to their own life situation that makes it possible to attribute a meaning to death. The previous interpretations indicate that being an older adult means to live in the increasingly apparent shadow of death. The past, present and future are intertwined in the fact that the temporal horizon of human existence is shrinking. This fact both enables and limits the possibility of being free to live out in an authentic manner the life that remains.

Hence, the awareness of death as the inevitable end of human existence incorporates existential concerns about authenticity and what it means to be human. Insight into the finiteness of life includes what de Beauvoir (De Beauvoir, 1947/1992, 1996) calls the ambiguity of human existence, which is to exist in order to die. This temporality of being human affects the whole structure of life. Ageing means that the shadow of death increases and the horizon of death comes closer, constantly present and inevitable. The vulnerability of human beings becomes particularly evident when one is faced with illness, either one’s own or that of someone to whom one is closely related. Serious illness can be frightening because it cannot be controlled. The search for relief may create the need to push all thoughts of death away, to displace it.

Making sense of living close to the finality of life, and still experiencing oneself as healthy and feeling well, therefore involves an intertwining of the displacement of death, whereby frightening feelings about old age and death are repressed, and the reconciliation of old age and death, whereby finality is regarded as a meaningful end to a rich life. Not until the displacement of death is intertwined with the reconciliation of death can the finality of life be accepted to the extent that the awareness of death can become a liberation and a companion late in life. To remain in displacement means to avoid working through frightening thoughts, which results in discomfort, death anxiety and the loss of meaning in life. Thus, when a person experiences health and well-being in old age, their awareness of the finality of life is ambiguous, since reconciliation and displacement exist side by side in a way that is both discomfiting and attractive. The intertwining of displacement and reconciliation appears to make it possible to internalize an intense desire to continue to live a meaningful life as it is at the moment, as long as that life does not deprive one of everything that makes it worth living.

## Discussion

In a lifeworld hermeneutic study, the process of interpretation and understanding lacks both a beginning and end. According to Radnitzky (1070), the hermeneutic spiral always lacks a zero point where the process of understanding begins, and where it definitely ends we can only guess. Thus, our interpretations only constitute our stay in a hermeneutical spiral (cf. Ödman, 2007). Vattimo’s idea that hermeneutics is about thoughts that are contrary to strong thinking with absolute claims of truth (Vattimo, 1988) makes it fair to believe that our interpretations constitute a few of many interpretative possibilities. The idea that each interpretation invites to other interpretations is significant for hermeneutics (cf. Ödman, 2007; Radnitzky, 1970; Vattimo, 1988). This approach is less common in descriptive qualitative methods, for example content analysis, which usually present findings as true, condensed abstractions.

We hope that this study help caregivers reflect on something that concerns us all, regardless of age: the finality of life. The issue of validity has been about ensuring, in the various ways mentioned in the method description, that there is a strong relationship between data and interpretation.

The findings of the present study are discussed further using ideas from the fields of existential philosophy and caring science to develop a basis for caring practice. Questions about the meaning of life, particularly in relation to ageing and death, are common themes in the philosophy of existence (De Beauvoir, 1947/1992, 1996; Heidegger, 1962/2008; Sartre, 1943/2018). Empirical research has contributed to our knowledge of several aspects of this matter, such as death anxiety in old age (Fortner & Neimeyer, 1999; Scott & Weems, 2013), the relationship between death anxiety and personal anxiety (Depaola et al., 2003) and how the finality of life is perceived by people of different age groups (Chopic, 2017; Maxfield et al., 2014).

In the present study, older adults were invited to talk about the fact that life will inevitably end. The participants estimated that their health was still good and spoke freely about death as our common destiny. The findings are arranged into five interpreted themes of meaning that are compared, abstracted and further interpreted into a comprehensive understanding, which should be considered the main finding of the study. The initial interpretations of death as a liberation and a companion in life appeared to be a motivating factor for taking full advantage of the life that remains. However, this requires that thoughts about the finality of life are not limited to frightening thoughts that must be repressed and displaced. Thus, a meaningful final stage of life requires that displacement be intertwined with reconciliation.

The comprehensive understanding was inspired by Simone De Beauvoir, (1947/1992, 1996). To Jean-Paul Sartre’s (1943/2018) ideas regarding how the finality of human existence grants meaning to life as a whole, she added statements about death making life meaningful and how that meaning might be. de Beauvoir means that older adults themselves must give their existence a meaning, asserting that older adults must cultivate their inner resources to bring meaning to their lives and make true connections with others. In younger days, people are occupied with ambition earning money and holding up a façade to satisfy oneself through the eyes of the other, instead of being true to oneself, which may prevent living with authenticity. Old age can, on the other hand, bring a certain freedom and even help nurture a questioning and challenging state of mind and living in an authentic way.

In the present study, such authenticity is connected to meaning that death can be liberating in the sense that death frees a person from suffering, but this does not apply to death in general. The death of a person to whom one is closely related can be worse than the idea of one’s own death. It also seems necessary not to completely avoid and distance oneself from thoughts about the finality of life to manage death anxiety.

Sartre (1943/2018) considers such a denial as living an inauthentic life, the sole purpose of which is to manage the ambiguity of life and death by keeping thoughts of death at a distance. According to the present study, it is when an older adult confronts the illness or death of a loved one, or one’s own illness, that they may realize their own vulnerability, which inspires them to live an authentic life. The results of our study reveal that when human beings reconcile themselves to the finality of life through faith, religion, and giving past and present life a meaning that can be brought into the future, this brings hope and the promise of continued life in one form or another after death. This reconciliation is comforting and means that life can be lived in meaningful relation to the finiteness of human existence.

With respect to the findings, the finality of life is also interpreted as a horizon of temporality, with reference to the title of Martin Heidegger’s magnum opus, *Being and Time*. According to Heidegger (1962/2008), being is essentially temporal because it only exists *between* being and not being, or being-towards-death. Accordingly, being is not temporal merely because it exists in time but rather because it is rooted in temporality, which is the original unity of future, past and present. It is a movement through a world that should be understood as a space of possibilities. In the present findings, the participants’ awareness of temporality was obvious in statements about our limited time on earth and the importance of taking advantage of the time we have at our disposal. It became additionally clear that being is rooted in time, along with an increased understanding of the nearness of death or, in the words of Heidegger, being-towards-death.

Rahm-Hallberg (2004) explores the existential matters found in the present study and finds further variations on the same theme. She concludes that older adults do not mind talking about their own death but want to feel alive until the very end. From a caring science perspective, health means feeling well and having the possibility of completing one’s life projects. The present study shows that it is important for older adults to have the opportunity to do what they want to do in life and not be dependent on others. This requires reconciliation with the fact that life will inevitably end. Thus, it is reasonable to regard reconciliation as an important aspect of health and well-being in old age.

Research shows that many older adults want to remain at home for as long as possible, since the home is closely linked with an older adult’s identity, integrity and way of living (Gillsjö et al., 2011). This raises the question of what it means to be a family caregiver and to care for a close family member in one’s home. Jarling et al. (2019) investigated how a person’s life situation is affected by the responsibility of caring for older relatives in their own homes in Sweden, which yielded interesting findings. Although the Swedish social service legislation is generous in supporting family care, the situation of family caregivers appears to resemble that of caregivers in countries without such legislation. Jarling et al. conclude that to ensure high-quality home care, those who perform family care should be granted the opportunity to express their concerns about their own life situations.

Our study finds yet another interesting aspect of this dilemma. The participants were keen that their children should not be burdened with the responsibility of caring for them when they may need care and support. This desire was so strong that death was regarded by some as relief and liberation from being a burden to one’s children. This may indicate a theme of not wanting to be a burden on one’s children. There is also a need for that particular relationship not to undergo the changes required to shift from parent to child, or carer and person receiving care. Informal family caregivers describe caring for a loved one as a responsibility that never rests, which paradoxically is both voluntary and non-chosen. In such responsibility, the previously close relationship as a husband, wife or child is affected in a way that makes it unequal and hard to manage, yet it is also a source of doing something meaningful (Jarling et al., 2019). These changed relationships may be seen as an ontological challenge that, according to the results of the present study, many older adults wish to avoid. Jarling et al. (2018) also highlight the complex issue of being cared for in one’s own home, which brings a sense of being forced to adapt to a caring culture that one cannot choose. When an individual feels unable to influence care, feelings are evoked of being exposed and forced into care relationships. In terms of caring science, and with the perspective of developing caring practices that are sensitive to such changed relationships, Galvin and Todres (2013) work about existential well-being, in which intersubjectivity and identity might be changed through needing to be cared for, could apply. They highlight mutual complementarity as an intersubjective well-being experience in which both kinship and belonging, as well as mysterious interpersonal attraction, are important. This may be more concrete if the older adult and their loved ones think through their relationships and together form a plan, in advance, for an eventual change to the relationship. This could be approached as something new in the relationship that enables a new direction in life, while retaining older elements that preserve the feeling that the relationship is still similar to what it was before. In preparation for such, health care professionals may have an important caring task to perform, even to healthy older adults, in order to prepare for future dependency namely, opening caring dialogues in which a changed intersubjective relationship is talked through with the goal of enabling well-being.

Although they may still be healthy, older adults might have health concerns that are taken care of by professionals in the health care system. These concerns may not be life-threatening but may still relate to the finiteness of human existence, making a caring dialogue favourable for continued health. In relation to the present findings and the discussion about changed intersubjective relationships, such dialogue requires a caregiver with the courage to realize that ageing means living in the increasingly apparent shadow of death. It is important not to attempt to avoid the fact that the temporal horizon is limited in old age. A caring science perspective founded on the theory of the lifeworld can provide an adequate knowledge base to develop a caring practice with sensitivity to such concerns (Dahlberg, 2011; Dahlberg, Todres, et al., 2008; Todres et al., 2007). According to Galvin and Todres (2013), a humanizing framework with a lifeworld approach is necessary to address health and illness at any age. They suggest that qualitative research, such as that conducted for the present study, has the potential to illuminate and guide directions for lifeworld-led health care by offering an existential view of human existence and well-being (Dahlberg, Todres, et al., 2008).

At the end of life, health can relate more to well-being than to relief from ill health in a strictly medical sense. According to Gadamer (1996, p. 77), to experience well-being, it is “enough to be able to say that one feels well, and to mean by that the ability to be completely involved in something else, in whatever else it is that one wants to do”. In the field of caring science, Dahlberg (2011) suggests that health and well-being are about being able to go through with one’s life projects. Thus, the findings of the present study appear to be consistent with both Gadamer’s philosophy and the principles of caring science. To reconcile with death may be an important way of attributing meaning to the final part of life and experiencing well-being. If so, caring dialogues can provide security and foster acceptance. The ambiguity of life and death, as well as the intertwining of reconciliation and displacement, may also draw attention to one’s life projects. Dahlberg and Segesten (2010) also suggest that well-being is intertwined with existential vitality. The results of the present study show that it is reasonable to assume that existential vitality is of obvious importance to being able to proceed with final stage of lifeas one wishes. When older adults do not experience existential vitality, their lives can become meaningless and they may begin to become reconciled with death. Thus, creating the conditions for well-being when vitality is lost is a challenge for health care professionals. Suffering may be overwhelming without such conditions, which can make life difficult to manage (Eriksson, 2006). The present study shows that suffering may occur when thoughts of death are so frightening that they make reconciliation impossible.

Nevertheless, the findings also show that healthy older adults seem to have opportunities for well-being. The finiteness of life is ambiguous, since the present study shows that reconciliation and displacement exist side by side in an existence that is both disturbing and attractive and in an intense desire to live and to experience all the possibilities of life here and now. Hence, in order to facilitate well-being, a lifeworld-led approach (Dahlberg, Todres, et al., 2008) that involves a caring dialogue with older adults and directly focuses on the individual person’s life situation is necessary. The present study emphasizes, for example, that thoughts about a worthy end of life include being independent and able to live one’s life as wanted and retain agency over one’s own life for as long as possible. In a recent study, Palmér et al. (2019) stress that thinking about one’s own death does not seem to be as frightening as the risk of being forced to live an alienated and unworthy life. An unworthy life seems to relate to the loss of beloved people, loss of bodily health, loss of memory and other cognitive functions and, perhaps most detrimental, loss of dignity.

To further develop the philosophical basis for understanding the needs of older adults through caring dialogue, it is necessary to consider the concept of the lived body. Thus, the lived body could be understood as a subject/object body—in other words, the body is both a subject and an object. Related to the present study, ageing is both a subjective and objective, an experiential and biological, entity. A caring dialogue must focus on ageing as both a lived experience and a biological fact, which are intertwined with each other.

The existential theory of the lived body, developed by the French philosopher Merleau-Ponty (1962/2002), can serve as a foundation for regarding a dialogue as a shared existence in which human beings live intentionally. Merleau-Ponty states that human existence is founded in the lived body and characterized by a body-mind-world unity. The lived body describes the whole of an individual’s existence, which involves the interaction between oneself, others and the world. As humans, we exist in an intersubjective manner, immersed with those meanings that life is full of.; Bullington (2013) takes this philosophical idea further in developing the meaning of the lived body for holistic care. She describes the importance of adopting a phenomenological (lifeworld-led) perspective, reflecting on the lived body and the natural attitude (the taken-for-granted) to create the possibility of holistic dialogue with a patient—one that takes into account their unique way of being older as both experiential and biological, subjective and objective. Merleau-Ponty, 2002 suggests:

In the experience of a dialogue, there is constituted between the other person and myself a common ground; my thought and his/hers are interwoven into a single fabric, my words and those of my interlocutor are called forth by state of the discussion, and they are inserted into a shared operation of which neither of us is the creator. (p. 353)

A phenomenological, lifeworld-led approach to a caring dialogue means that both the health care professional and the older adult are active and create the conversation in an intersubjective manner so that meaning is generated in between the health care professional and the patient (Todres et al., 2007). In order for professionals to understand their part in improving an individual’s health, it is necessary for them to adopt a phenomenological, lifeworld-led attitude that is open and sensitive to older adults’ existential questions. Such openness could be concrete and ask questions like, “Can you please tell me your thoughts about death and the finality of life?” and “What is a worthy end of life for you, and how could caring activities be performed in order to enhance your ability to live out life feeling well?”

## Conclusions

Awareness of the finality of life means living in an ambiguity whereby death is the final horizon of life. For an older adult, it may be important to talk about this with a person who dares to listen and wants to understand. Therefore, high-quality health care should involve readiness for a caring dialogue that focuses on such issues. The existential dimensions of life, such as temporality, freedom and the ambiguity of displacement and reconciliation, are important to consider when confronting the finality of life. An existential caring science based on a lifeworld perspective in research on existential matters, as well as in the care of older adults, can stimulate the further search for meaning, not only in relation to the finality of life but to the whole of life.
